# Health and environmental impacts of sachet water consumption and sachet plastic waste in Nigeria: a scoping review

**DOI:** 10.1186/s12889-026-28682-6

**Published:** 2026-07-28

**Authors:** Hilary I. Okagbue, Nnenna U. Kwaghshir, Success C. David, Eyitayo A. Lawal, Olanma Okezie-Okafor, Deborah Kolawole, Sunday Nto, Sunday Atobatele, Sidney Sampson

**Affiliations:** 1 Sydani Institute for Research and Innovation, Sydani Group, Abuja, Nigeria; 2 Sydani Initiative for International Development, Sydani Group, Abuja, Nigeria

**Keywords:** Scoping review, Sachet water, Plastic waste, Water quality, Nigeria

## Abstract

**Introduction:**

Access to safe drinking water remains a major public health challenge in Nigeria, where sachet water is widely used due to limited piped water supply. Despite its importance, sachet water raises concerns regarding health risks and the environmental plastic waste burden. The aim of this review is to map existing evidence on the health impacts of sachet water consumption and the environmental consequences of plastic waste in Nigeria.

**Methods:**

A scoping review design was adopted for this review in line with the PRISMA-ScR guideline. Four databases were searched, which are PubMed, Scopus, AJOL and Google Scholar, for studies on sachet water, plastic waste, health impacts, and environmental impacts. The search was limited to studies published between 2016 and 2026. Inclusion criteria encompassed primary studies of communities in Nigeria exposed to sachet water consumption or sachet water-related plastic waste. Three reviewers independently screened titles/abstracts and full texts and extracted data. A narrative synthesis was conducted due to methodological heterogeneity.

**Results:**

Of the 1,940 records identified from four databases and screened following PRISMA-ScR guidelines. After screening and eligibility assessment, 20 studies met the inclusion criteria across Nigeria. Thirteen studies reported health outcomes, including widespread microbial contamination (*E. coli*, *Salmonella*, *Shigella*), elevated heavy metals (Pb, Cd, Hg, As), and detection of phthalates, with several exceeding WHO drinking-water guideline limits. Three studies reported environmental outcomes, including microplastic pollution and soil/agricultural disruption. Evidence from the review indicates that sachet water safety is influenced by contamination at production and along the supply chain, with environmental conditions such as heat and sunlight contributing to chemical migration and degradation.

**Conclusion:**

This review found that sachet water in Nigeria is associated with microbial and chemical contamination, posing important public health risks. In addition, sachet plastic waste has been identified as a contributor to flooding, drainage blockages, and microplastic pollution. These findings highlight the need for stronger regulation and an improved waste management system.

**Supplementary Information:**

The online version contains supplementary material available at https://doi.org/10.1186/s12889-026-28682-6.

## Background

Access to safe drinking water is a basic requirement for health and well-being, yet it remains a challenge in many parts of the world [50]. Although the global coverage of access to safe drinking water increased from 68 to 74% between 2015 and 2024 [53], 2.1 billion people still lack access to safely managed drinking water, with 302 million people still relying on unimproved water sources, particularly in urban areas in low-income countries [51]. For water to be considered safe and drinkable, it must be free from pathogenic microorganisms, physical impurities and debris, as well as chemical contaminants such as toxins, carcinogens, radionuclides, and other health risks. In developing countries, people lack access to potable water and even where there is access, water remains unfit for human consumption and domestic use [23].

In response to these challenges, international health bodies have classified drinking water sources by safety and construction quality, differentiating between improved and unimproved sources. Improved drinking water sources are sources that, by design and construction, can deliver water safely [11]. These sources include piped household water connection, public standpipe, borehole or tube well, protected well, protected spring water, rainwater collection, package or delivered water, water distributor, water kiosks, bottled water, and sachet water [11].

In many low- and middle-income countries, especially in West Africa, where access to piped water is limited and unreliable, residents depend on alternative sources of water, which are commercially produced, treated and distributed in sealed containers such as plastic bottles and small sachets [48]. In Nigeria, packaged sachet water is typically packaged in polyethylene (commonly referred to as polyethene), a type of plastic and is locally referred to as pure water. It has become the major source of drinking water for people due to low cost, portability and widespread availability [5, 55].

Since its introduction in the 1990 s, the production and consumption of sachet water have rapidly expanded in Nigeria, mostly due to the nation's deficiencies in public water infrastructure, rapid urbanisation, and increased demand for affordable drinking water [36]. Sachet water is an essential source of potable water for millions of Nigerians, especially for those living in urban and peri-urban areas where pipe-borne water supplies are unreliable or unavailable [43]. Despite its contribution to improving access to drinking water, concerns regarding the microbiological and chemical quality of sachet water and the environmental burden associated with discarded sachet water have been documented for several decades, highlighting the need for continued evaluation of its health and environmental implications.

Sachet water contamination has been associated with an increased risk of waterborne diseases such as diarrhoea, cholera, and typhoid [35]. In contrast, bottled water showed little to no contamination with these infectious microorganisms [48]. In addition to microbial risks, evidence has established the presence of heavy metals such as lead, cadmium, copper, and nickel above the recommended thresholds in sachet water, which can accumulate in human tissue and cause chronic illness [41].

Beyond its health implications, the mass consumption of packaged water has generated a substantial and growing plastic waste burden with serious environmental consequences. Over 60% of sachet-related plastic pollution in Nigeria is reported to be due to sachet water plastic waste [47], which is attributed to the fact that approximately 7 in 10 Nigerians consume at least one sachet water daily, thereby resulting in an estimate of 50 to 60 million discarded sachets daily [8].

The indiscriminate littering of sachet and empty bottle waste along the streets, in gutters, in market spaces, in school compounds, and in other places [33] leads to blockages in drainage systems [55] and flooding [34]. Furthermore, this indiscriminate disposal of plastic waste clogs domestic sewage systems, chokes aquatic animals and affects soil quality as they are made of non-biodegradable material which may not degrade even after years [34, 55]. Even when burnt, plastic waste produces harmful gases such as carbon monoxide, nitrogen oxides, methane, and sulphur dioxide, contributing to air pollution and global warming [13].

While sachet water has expanded access to drinking water for many households, especially low-income households, there are increasing concerns about the public health and environmental burden of sachet water consumption in Nigeria. Despite the urgency of these challenges, there is little documented evidence on the health impacts of consuming sachet water and the environmental impacts of the indiscriminate disposal of the resulting waste. Therefore, this review aims to synthesise evidence on the health impacts of sachet water use and the environmental impacts of the plastic waste generated in Nigeria. This review’s findings will contribute directly to the achievement of Sustainable Development Goals (SDGs) 6 and 12, which focus on clean water and sanitation and responsible consumption and production. It will contribute indirectly to SDGs 14 and 15, which centre on protecting aquatic and terrestrial ecosystems from growing plastic pollution.

## Material and method

### Study design

This study adopted a scoping review design to map the existing evidence on the health and environmental impact of sachet water consumption and associated plastic waste. This design was selected as it provided a wide scope of a specific field. A scoping review is considered appropriate due to the broad and exploratory nature of the research topic and the need to identify key concepts, types of evidence and knowledge gaps [7]. This review was carried out based on the methodological guidance provided by the Joanna Briggs Institute (JBI) for scoping reviews. Thus, ensuring a systematic, transparent and reproducible process in identifying and synthesising evidence across several study designs.

The review is developed in line with the stages of the JBI framework, which includes (i) research question identification; (ii) identification of relevant studies; (iii) selection of eligible studies; (iv) data extraction; and (v) summarisation and reporting of results. This enables structured extraction of detailed information from the literature.

The review was reported in line with the PRISMA-ScR checklist, which ensured methodological rigour and transparency [45]. The use of this guideline supports detailed reporting of the search process, study selection and findings synthesis. The review protocol was not registered prior to conducting this scoping review,however, the review methods were developed following the Joanna Briggs Institute methodological guidance for scoping reviews. No critical appraisal of included studies was conducted, as this scoping review aimed to map the available evidence rather than assess methodological quality, in line with Joanna Briggs Institute guidance for scoping reviews.

### Review framework

The review followed the JBI methodological framework as described in the study design [40]. The review questions were developed using the Population-Exposure-Outcome (PEO) approach, which is applicable to environmental and public health research. In this review, the population was made up of communities, households, and persons in Nigeria who consumed sachet water, as well as persons exposed to sachet water-related plastic waste using environmental proximity and contamination pathways. Moreover, exposure was defined as the consumption of sachet water and the production, disposal and accumulation of plastic waste. In addition, the outcome includes both health impacts and environmental impacts. Guided by this approach, the research question examined the evidence on health and environmental outcomes associated with sachet water consumption and sachet water-related plastic waste in Nigeria. The PEO framework ensured that the review question was clearly defined, aligned and broad in capturing the available evidence while still maintaining focus on health, environment and governance in relation to sachet water use and plastic waste in Nigeria.

### Eligibility criteria

The eligibility criteria in this review were developed based on the PEO framework and guided by the methodological recommendations of the JBI framework. Articles were included based on their importance to the study population, exposure and outcome, as well as their focus on Nigeria (Table 1). The review eligibility criteria were designed to ensure that only studies that were important to the review objectives and Nigeria were included. Thus, restricting inclusion to English-language and Nigeria-focused studies was required due to feasibility constraints and ensures accurate interpretation of findings.

**Table 1:** Eligibility criteria of included studies

	**Inclusion**	**Exclusion**
Population	Studies that included individuals, households, or communities in Nigeria exposed to sachet water consumption or sachet water-related plastic waste through environmental or occupational pathways	Studies not involving human populations or not focused on Nigeria. Studies without Nigeria-specific data
Exposure	Studies that involved sachet water consumption and/or production, disposal, or accumulation of polyethylene sachet waste	Studies where sachet water is not a primary exposure (bottled water-only studies)
Outcome	Studies that included microbial, chemical, or physical health outcomes linked to sachet water consumption (infections, toxicity, gastrointestinal illness)	Studies not reporting any health outcomes related to sachet water exposure
	Studies that assessed environmental impacts such as plastic pollution, soil and water contamination, drainage blockage, or ecosystem degradation linked to sachet water waste	Studies not assessing the environmental impacts of sachet water-related waste
Other Criteria	Peer-reviewed quantitative, qualitative and mixed-method studies published in English, reporting complete and finalised findings	Studies in other languages, unfinished studies, conference abstracts, editorials, and commentaries

### Data source and search strategy

A detailed and systematic search strategy was developed to identify relevant literature on the health and environmental impacts of sachet water consumption and associated plastic waste in Nigeria. Several data sources were used to ensure broad and comprehensive coverage of peer-reviewed literature, as recommended by JBI [40]. Searches were conducted across the following databases: PubMed, Scopus, Google Scholar and African Journals Online (AJOL). These databases were selected because of their extensive coverage of public health, environmental science and relevance to water quality and environmental pollution [7].

The search term combined keywords using Boolean operators (AND, OR), which ensures detailed retrieval of relevant studies. The key search terms included combinations of “sachet water,” “pure water,” “plastic waste,” “polyethylene bags,” “health effects,” “environmental pollution,” and “Nigeria.” Search strings were adapted for each database to reflect indexing differences. In this review, the terms “sachet water,” “pure water,” and “packaged water” were treated as synonymous and used interchangeably, as they are commonly used in the Nigerian context to describe commercially packaged drinking water. This ensured comprehensive capture of all relevant studies regardless of the terminology used.

To ensure comprehensiveness, the reference lists of all included studies were manually screened to identify important publications not captured in the database searches. All searches were limited to studies published in English, as English is the primary language of academic publication in Nigeria and most peer-reviewed research in public health and environmental sciences from the region is published in English. Therefore, this restriction is unlikely to have introduced significant language bias while ensuring accurate interpretation of findings. Searches were conducted between 22 and 28 April 2026.

In this review, a PubMed search strategy combined terms related to sachet water, plastic, health and environmental outcome using Boolean operators. The complete PubMed search string is presented below as an example. The corresponding search strategies adapted for the other databases are provided in the supplementary file.

(("sachet water" OR "packaged water" OR "drinking water") AND (plastic* OR waste OR pollution OR contamination) AND (health OR environmental) AND (Nigeria OR Africa))

### Study selection

Identified articles were imported into Rayyan to ensure centralised management and the removal of duplicates. Screening was then carried out in two sequential stages by 3 independent reviewers (EL, SD & DK). In the first stage, titles and abstracts were screened based on the eligibility criteria, and in the second stage, full-text articles were retrieved and independently assessed for eligibility. Discrepancies among the reviewers during screening were resolved through discussion and consensus; in instances where consensus was not reached, a majority decision was used. The study selection process was conducted in accordance with guidance from the Joanna Briggs Institute and reported using the PRISMA-ScR framework. A flow diagram was used to summarise the screening and selection process (Fig. 1).

**Fig. 1:**
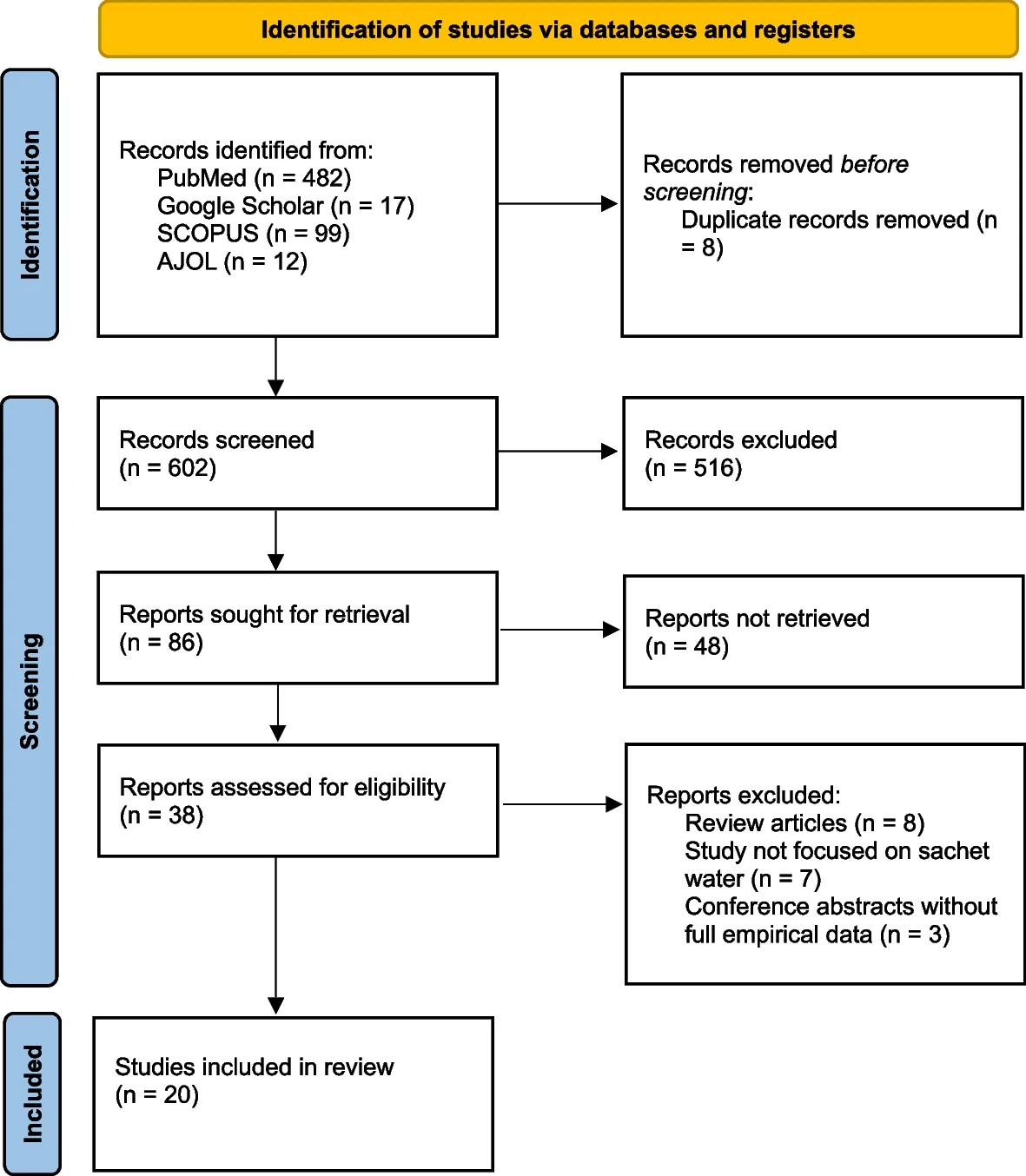
PRISMA flowchart

### Data extraction

Data extraction was carried out using a standard data chart form created in Microsoft Excel in line with the JBI guidance for JBI scoping review. The form was formulated to ensure consistent and systematic extraction of important information from all included studies. The charting form was pilot tested on a subset of the included studies before full data extraction and refinement, where needed, to improve the clarity and consistency among the reviewers [27]. Data extraction was carried out independently among all reviewers, and discrepancies were resolved using discussion and consensus. The information extracted included author(s), year of publication, study location, study design, population characteristics, type of exposure, health outcome(s) assessed, environmental outcome(s) assessed, key findings, and policy implications. The data extraction process enabled reviewers to modify and update the charting. Thus, the extracted data were synthesised descriptively and organised into themes that are in line with the review objectives.

### Data analysis and synthesis

Data analysis and synthesis were conducted using a descriptive and thematic approach consistent with methodological guidance for scoping reviews. Extracted data were summarised to map the breadth, nature, and distribution of evidence relating to the health and environmental impacts of sachet water consumption and sachet water-related plastic waste in Nigeria. Descriptive analysis was used to summarise the characteristics of the included studies. The findings were presented using tables and narrative summaries to provide an overview of the available evidence. A thematic synthesis approach was employed to identify and organise repeated patterns and concepts across the included studies. The findings were grouped into major thematic domains that are in line with the review objectives and research questions. Subthemes were developed inductively based on patterns emerging from the extracted data.

The synthesis process was interpretive, which enabled refinement of themes as familiarity with the literature increased, consistent with scoping review methodology. No meta-analysis or quantitative pooling of results was undertaken due to the heterogeneity of study designs, outcome measures, and methodologies among the included studies. The results were reported narratively and supported with summary tables and thematic categorisations to facilitate interpretation of the evidence base and identification of knowledge gaps relevant to future research and policy development.

## Result

A total of 1,940 articles were retrieved from four peer-reviewed databases (PubMed, Scopus, Google Scholar and AJOL) and screened based on the PRISMA-ScR guidelines. After the removal of duplicates, 602 records remained for title and abstract screening. During this stage, 516 records were excluded because they did not meet the eligibility criteria, primarily as they were not focused on sachet water consumption or sachet water-related plastic waste, were not conducted in Nigeria, did not report relevant health or environmental outcomes, or were not primary peer-reviewed studies. Eighty-six full-text articles were sought for retrieval, of which 38 were assessed for eligibility. Following full-text assessment, 20 studies met the inclusion criteria for this review (Fig. 1 and Supplementary Data 2).

The 20 studies were conducted in Nigeria, with the highest number in the Southwest (*n* = 5). Health outcomes were reported in 13 studies, including microbial contamination and waterborne disease (*n* = 5), heavy metal exposure (*n* = 4), chemical migration from packaging materials (*n* = 2), and multi-risk health outcomes (*n* = 2). However, only three studies directly assessed environmental outcomes, reporting evidence related to microplastic contamination (*n* = 2) and soil/agricultural disruption (*n* = 1).

### Characteristics of included studies

20 studies met the inclusion criteria and spanned the period from 2016 to 2026. Study designs included experimental laboratory-based studies (*n* = 13), cross-sectional surveys (*n* = 5), mixed-method studies (*n* = 1), and qualitative studies (*n* = 1). Geographic coverage included North central (*n* = 2), Northeast (*n* = 4), Northwest (*n* = 3), Southeast (*n* = 4), South-South (*n* = 2) and Southwest (*n* = 5). States represented across the geographic region are presented in Table 2.

**Table 2:** States represented across the geographic region

Geographic location	States
North Central	Nasarawa, Abuja, Kwara
North-East	Adamawa, Gombe, Borno, Taraba
North-West	Kebbi, Jigawa, Kano
South-East	Enugu
South-West	Oyo, Lagos, Ondo
South-South	Cross-River

The characteristics and principal findings of the included studies are summarised in Supplementary Data 2

### Synthesis of findings

#### Health outcomes of sachet water consumption

##### Microbial contamination and waterborne disease

Five studies assessed the quality of sachet water in different Nigerian settings [4, 9, 14, 25, 37]. The studies sampled commercially sold sachet water from streets, markets, or retail outlets. All five studies reported detecting indicator organisms or pathogens in at least some sachet water brands. *Escherichia coli* was identified in multiple brands in three studies [4, 25, 37], with total coliform counts exceeding national and WHO drinking-water guideline values. Other reported organisms included *Salmonella* spp. and *Shigella* spp. [9, 14]. In one study, only a minority of brands met acceptable microbiological standards [37]. Some studies also reported multidrug-resistant isolates in contaminated sachet water [4, 9].

##### Heavy metal exposure

Four studies evaluated heavy metal concentrations in sachet water [3, 17, 41, 54]. These studies analysed metals such as Pb, Cd, As, Hg, Fe, Mn and others using standard laboratory methods. Study characteristics are shown in Table 3.

**Table 3:** Heavy metal exposure

Study ID	Heavy metals present
[17]	Manganese (Mn), Cadmium (Cd), Mercury (Hg), Lead (Pb), Aluminum (Al), Iron (Fe)
[41]	Lead (Pb), Mercury (Hg), Nickel (Ni), Cadmium (Cd), Arsenic (As)
[54]	Lead (Pb), Copper (Cu), Cadmium (Cd), Chromium (Cr), Iron (Fe)
[3]	Iron (Fe), Copper (Cu), Manganese (Mn), Cadmium (Cd), Mercury (Hg), Lead (Pb), Arsenic (As)

All four studies detected at least one heavy metal in sachet water samples. Lead and cadmium concentrations exceeded the WHO drinking-water guideline limit in some brands in three studies [17, 41, 54]. Elevated manganese and iron levels were also reported above permissible limits in selected samples [3, 54]. One study found that all sampled brands contained Pb above recommended thresholds [17]. The authors generally concluded that chronic consumption of such water may pose toxicological risks.

##### Chemical migration from packaging materials

Two studies investigated chemical migration from plastic sachet packaging into water [12, 22]. Both examined phthalates and related organic compounds, including the effects of storage conditions (sunlight exposure, ambient temperature). Details are summarised in Table 4.

**Table 4:** Chemical migration from packaging materials

Study ID	Phthalates/ Organic compounds present
[22]	Dimethyl phthalates (DMP) Diethyl phthalates (DEP) Dibutyl phthalates (DBP)
[12]	Dimethyl phthalates (DMP) Dibutyl phthalates (DBP) Butyl benzyl phthalates (BBP) Mono-2-ethyl-hexyl phthalates (MEHP) Phthalic acid decyl hept-2-yl ester (PADHE)

Both articles reported detectable levels of phthalates in sachet water, with higher concentrations compared to bottled water in one study [22]. Concentrations increased with prolonged storage under sunlight or elevated temperature [12]. In some cases, measured values approached or exceeded health-based guideline values for drinking water.

##### Multi-risk health outcomes

Two studies examined the multi-risk health outcomes associated with sachet water consumption [31, 42]. Both articles highlighted that sachet water safety cannot be considered solely in terms of either microbiological or chemical quality. Instead, they pointed to concurrent risks arising from heavy metal contamination, suboptimal physicochemical characteristics, and, in some cases, potential microbial hazards linked to source water and environmental conditions.

Shehu [42] reported that lead concentrations exceeded WHO drinking-water guideline values across all sampled sachet water brands, while mercury, cadmium, and arsenic also exceeded permissible limits in several samples. These co-occurring heavy metal exposures were associated with multiple chronic health risks, including neurological impairment, renal dysfunction, cardiovascular disease, and developmental toxicity.

Musa et al. [31] identified concurrent physicochemical and microbiological concerns in sachet water and its source waters. Some samples exceeded acceptable hardness thresholds, while acidic conditions recorded in source boreholes were noted as potentially favouring microbial proliferation, which poses a risk of waterborne diseases, including typhoid and cholera, attributed to contaminated source water and surrounding environmental activities.

#### Environmental outcomes of sachet water and plastic waste

##### Microplastic contamination

Two studies investigated microplastic contamination associated with sachet water plastic waste [13, 30]. Both reported discarded sachet water packaging as a significant source of microplastic pollution in urban and environmental settings. One of the studies reported poor waste disposal and high densities of microplastic fragments and films in urban drainage channels, contributing to pollution, flooding, and microplastic contamination. [6].

Donuma et al. [13] documented microplastic contamination across terrestrial and aquatic environments, identifying single-use sachet water packaging as a dominant source of small-sized polyethene fragments. This provided a surface for biofilm formation and adsorbs heavy metals and organic pollutants, indicating a potential role as contaminant vectors within ecosystems.

##### Soil and agricultural disruption

One study reported the interaction between sachet plastic waste and agricultural soils [15]. Contaminated soils exhibited reduced porosity, impaired water infiltration, and lower crop performance compared with uncontaminated controls. Elevated concentrations of cadmium and lead were also recorded in microplastic-rich soil layers, suggesting that plastic particles may facilitate heavy metal accumulation and mobility in agricultural soils.

#### Environment-health linkages associated with sachet water system

Four studies examined the relationship between environmental exposure conditions, plastic degradation, waste disposal practices and potential human health risks associated with sachet water systems [6, 21, 29, 46]. Prolonged sunlight exposure of sachet water resulted in increased toxic metal concentrations and microbial contamination, indicating that environmental storage conditions may significantly compromise sachet water safety [46]. Ultrasound radiation and environmental heat exposure contributed to polyethene packaging degradation, with potentially harmful compounds, including hydrocarbons and benzene derivatives, released into sachet water contents under varying exposure conditions [21]. Furthermore, widespread indiscriminate disposal of sachet water plastics contributed to environmental pollution and poor waste management, which posed health risks from decomposition [6]. Finally, Malgwi et al. [29] identified elevated manganese concentrations in some sachet water brands, attributing this partly to source water characteristics, which may influence sachet water chemical quality and associated neurological health risks.

## Discussion

This scoping review synthesised the available evidence on the health and environmental impact of sachet water consumption and associated plastic waste in Nigeria. The findings were dominated by laboratory-based studies evaluating water quality, with most studies reporting health outcomes, particularly microbial contamination, heavy metal exposure, and chemical migration from plastic packaging materials. In contrast, only three included studies directly assessed environmental outcomes, reporting findings related to microplastic contamination and soil degradation. Collectively, the evidence indicates that research on the health implications of sachet water in Nigeria is considerably more developed than research on its environmental consequences.

The most consistent finding across the included studies was the detection of microbial contamination, including *Escherichia coli, Salmonella* spp.*, and Shigella spp*, corroborating prior studies on the microbiological quality of packaged drinking water [1, 44]. The occurrence of these microorganisms suggests lapses in water treatment, production hygiene, and regulatory oversight during sachet water production. Similar observations have been reported in other sub-Saharan African countries, where packaged drinking water has also been found to exceed WHO microbiological guideline values [20, 26]. Collectively, these findings suggest that microbial contamination of packaged drinking water is not unique to Nigeria but reflects broader regional challenges relating to water treatment infrastructure, production practices, and regulatory enforcement.

Beyond microbial contamination, the review consistently identified chemical contamination arising from both source water and plastic packaging. Heavy metals, including lead, cadmium, arsenic, and mercury, were reported in several included studies, with some concentrations exceeding recommended guideline values. Similar contamination profiles have been described in other low- and middle-income countries where groundwater serves as an important drinking water source and is affected by industrial activities, mining, and indiscriminate waste disposal [32]. The repeated detection of these metals across multiple Nigerian studies suggests that contamination is unlikely to be confined to isolated locations and may reflect broader environmental and regulatory challenges affecting groundwater quality. Comparable findings have also been reported in Ghana and Bangladesh, where groundwater-based drinking water systems remain vulnerable to both natural and anthropogenic contamination [2]. Differences in contaminant concentrations across studies may reflect variations in sampling periods, hydrogeological characteristics, and analytical methods.

The review also found that plastic packaging itself may contribute to chemical exposure. Detection of phthalates and other plasticisers in sachet water supports previous evidence demonstrating migration of endocrine-disrupting chemicals into drinking water, particularly following prolonged exposure to heat and sunlight [19, 24]. The tropical climatic conditions in Nigeria, coupled with prolonged outdoor storage and transportation of sachet water, may accelerate polymer degradation and increase chemical migration compared with temperate settings. These findings are important because chronic phthalate exposure has been associated with reproductive disorders, hormonal dysregulation, breast and testicular cancers, and neuroendocrine dysfunction [49].

Taken together, the findings demonstrate that sachet water consumers may be exposed to multiple contaminants simultaneously rather than isolated hazards. While microbial contamination presents an immediate risk of infectious disease, heavy metals and plastic-derived chemicals pose chronic health risks through cumulative exposure, including neurological impairment, renal dysfunction, developmental toxicity, endocrine disruption and carcinogenicity [52]. One included study estimated that although overall non-carcinogenic risks were generally low to moderate among adults, the Total Hazard Index reached moderate levels among infants [28], highlighting the greater vulnerability of young children in households where sachet water is routinely used for infant feeding and preparation of complementary foods. Similar patterns of multiple contaminant exposure have also been described in Bangladesh, where drinking water systems were characterised by the co-occurrence of microbial, heavy metal and chemical contaminants [38]. This suggests that cumulative exposure may represent a broader structural challenge affecting drinking water systems operating within resource-constrained settings..

Another significant insight from this review is that sachet water consumers in Nigeria do not face isolated singular risks; rather, they face a cumulative burden that arises from the co-occurrence of microbiological, heavy metal and chemical contamination in the same product that they consume daily. This multi-contaminant co-occurrence pattern is not unique to Nigeria alone, as a systematic review of drinking water quality in Bangladesh reported the presence of heavy metals, microbial contaminants and chemical pollutants [38]. This notes that it is a structural feature of water systems in low-income countries operating under inadequate infrastructure rather than a locally specific phenomenon.

Understanding why sachet water quality varies is equally important as documenting the contaminants themselves. The evidence mapped in this review suggests that three interacting factors influence sachet water quality: source water quality, production conditions, and post-production storage and distribution. Water source is the primary upstream determinant, with several included studies reporting that producers obtained groundwater from boreholes located close to markets, motor parks and mechanic workshops, increasing the likelihood of microbial and chemical contamination before treatment. Similar observations have been reported in Ghana, where groundwater characteristics influenced the physicochemical quality of sachet water [10]. In addition, evidence from the included studies revealed deficiencies in production infrastructure and organisational practices within some factories, suggesting that regulatory registration alone does not consistently ensure product quality. Comparable findings from Ghana demonstrated that inconsistencies in chlorination, ultraviolet irradiation and reverse osmosis contributed to residual contamination despite formal regulatory oversight [1].

Sachet water safety is not a static property fixed at the point of production; it is a dynamic one that degrades along the supply chain. Our review findings indicated that prolonged sunlight exposure increases toxic metal concentrations and drives physicochemical deterioration. Evidence shows increased concentrations of metals and other contaminants in sachet and bottled water exposed to prolonged sunlight [18]. This deterioration might be associated with heat-induced migration of packing materials during storage and transportation. The sachet water a consumer purchases from a vendor after storage may therefore be considerably more dangerous than the water that left the factory, and this impact is entirely invisible to current regulatory assessments.

Compared with the substantial evidence relating to health outcomes, environmental evidence was considerably more limited, with only three included studies directly assessing ecological impacts. Nevertheless, the available evidence suggests that improper disposal of sachet water plastics contributes to environmental pollution through drainage blockage, microplastic contamination and deterioration of soil quality. Two included studies identified discarded sachet water packaging as a major contributor to microplastic accumulation in drainage systems and surrounding terrestrial environments, with implications for urban flooding and environmental pollution. These findings are consistent with broader evidence demonstrating the persistence of plastic waste and its fragmentation into microplastics within terrestrial and aquatic ecosystems [55].

Only one included study investigated the effects of sachet water plastics on agricultural soils, reporting reduced soil porosity, impaired water infiltration, poorer crop performance, elevated heavy metal concentrations and increased abundance of multidrug-resistant bacteria in microplastic-contaminated soils [15]. These findings are consistent with broader evidence demonstrating that microplastic accumulation can alter soil physicochemical properties and disrupt biogeochemical cycling, thereby reducing nutrient availability and agricultural productivity [39]. The same study also suggested a possible association between microplastic-contaminated soils and increased abundance of antibiotic-resistant bacteria, indicating a potential environmental pathway that warrants further investigation. However, because only three Nigerian studies directly assessed environmental outcomes, these findings should be interpreted cautiously and should not be considered representative of the overall environmental burden associated with sachet water plastic waste. Additional Nigerian studies are required before broader conclusions can be drawn regarding its contribution to soil degradation, food security or antimicrobial resistance.

While none of the included studies in this review directly assessed animal health outcomes, the pathways are well-documented in the global microplastics literature: exposure can cause physical harm, digestive tract damage, endocrine disruption, and oxidative stress, with microplastics also functioning as vectors for heavy metals and organic pollutants released into animal tissues upon ingestion.

### Implications for policy, regulation and public health practice

The evidence mapped in this review indicates that improving sachet water safety in Nigeria requires strengthening regulatory oversight across the production and distribution chain. The consistent reporting of microbial contamination, heavy metals and chemical migration across included studies supports the need for enhanced monitoring of source water quality, manufacturing processes, storage conditions, and post-market surveillance.

Strengthening enforcement of existing quality standards by the National Agency for Food and Drug Administration and Control (NAFDAC), particularly regarding routine inspections, product traceability, and mandatory labelling (including manufacturing dates, expiry dates, and batch numbers), could improve consumer protection and facilitate product recall where necessary.

Although only a small number of included studies assessed environmental outcomes, they consistently highlighted concerns relating to improper disposal of sachet water plastics, including microplastic contamination and disruption of soil quality. These findings suggest a need to strengthen plastic waste management strategies, improve waste collection systems, and promote responsible disposal and recycling of sachet plastics. In addition to strengthening waste management systems, improving public environmental awareness and encouraging responsible disposal practices may support efforts to reduce sachet water plastic pollution, as previous Nigerian studies have highlighted the importance of environmental consciousness and citizen responsibility in achieving environmental sustainability [16].

Given the limited environmental evidence identified in this review, additional Nigerian studies are needed to better understand the scale of ecological impacts and to inform future environmental policy. Future research should also examine the long-term consequences of sachet plastic waste across different ecological settings within Nigeria.

### Limitations of the review

This review also identified several important evidence gaps. First, the existing literature is dominated by laboratory-based assessments of water quality, with relatively few epidemiological studies linking exposure to sachet water to population-level health outcomes. Second, environmental evidence remains sparse, limiting understanding of the ecological consequences of plastic waste from sachet water across different regions of Nigeria. Third, geographical representation was uneven, with some geopolitical zones contributing substantially more evidence than others. Finally, relatively few studies examined post-production factors such as transportation practices, retail storage conditions, consumer handling or long-term exposure to contaminants. Addressing these evidence gaps through multidisciplinary research would provide a more comprehensive understanding of both the health and environmental implications of sachet water consumption in Nigeria.

## Conclusion

This scoping review found consistent evidence that sachet water consumed in Nigeria may be affected by microbial contamination, heavy metals, and chemical migration from plastic packaging, highlighting important public health concerns. Evidence on environmental impacts was comparatively limited but suggested that improper disposal of sachet water plastics may contribute to microplastic pollution and soil degradation. Overall, the review highlights the need to strengthen water quality regulation, improve plastic waste management, and expand research on the environmental consequences of sachet water consumption in Nigeria.

## Supplementary Information

Supplementary Material 1. Search terms.

Supplementary Material 2. Extracted Data.

## Data Availability

The datasets used are presented as supplementary materials.

## Abbreviations

AJOL: African Journals Online
AMR: Antimicrobial Resistance
BBP: Butyl Benzyl Phthalate
CDC: Centers for Disease Control and Prevention
DBP: Dibutyl Phthalate
DEP: Diethyl Phthalate
DMP: Dimethyl Phthalate
ECOWAS: Economic Community of West African States
JBI: Joanna Briggs Institute
NAFDAC: National Agency for Food and Drug Administration and Control
PADHE: Phthalic Acid Decyl Hept-2-yl Ester
PEO: Population–Exposure–Outcome
PRISMA: Preferred Reporting Items for Systematic Reviews and Meta-Analyses
PRISMA-ScR: Preferred Reporting Items for Systematic Reviews and Meta-Analyses Extension for Scoping Reviews
SDGs: Sustainable Development Goals
UV: Ultraviolet
WHO: World Health Organization
