# Supplementary material for: Health and environmental impacts of sachet water consumption and sachet plastic waste in Nigeria: a scoping review
Source: BMC Public Health. 2026 Jul 28;26:3048. doi: 10.1186/s12889-026-28682-6 (PMC13692890; doi:10.1186/s12889-026-28682-6)
Supplement: Supplementary file 1 [file 12889_2026_28682_MOESM1_ESM.docx]

**PubMed**

The PubMed database was searched from 22^nd^ to 24^th^ of April 2026 using the following search string below. The search was limited to studies published between 2016 and 2026 and restricted to English-language publications.

(("sachet water" OR "packaged water" OR "drinking water") AND (plastic* OR waste OR pollution OR contamination) AND (health OR environmental) AND (Nigeria OR Africa))

**Google Scholar**

The search was carried out using the search string from 24^th^ to 26^th^ of April 2026. The search was limited to the first 10 pages, which were screened for eligibility. Only studies published between 2016 and 2026 and written in English were considered.

[(("sachet water" OR "packaged water" OR "drinking water") AND (plastic* OR waste OR pollution OR contamination) AND (health OR environmental) AND (Nigeria OR Africa))](https://scholar.google.com/scholar?q=((%22sachet+water%22+OR+%22packaged+water%22+OR+%22drinking+water%22)+AND+(plastic*+OR+waste+OR+pollution+OR+contamination)+AND+(health+OR+environmental)+AND+(Nigeria+OR+Africa))&hl=en&as_sdt=0,5&scilib=1&scioq=((%22sachet+water%22+OR+%22packaged+water%22+OR+%22drinking+water%22)+AND+(plastic*+OR+waste+OR+pollution+OR+contamination)+AND+(health+OR+environmental)+AND+(Nigeria+OR+Africa)))

**SCOPUS**

The Scopus database was searched on 28 April 2026 using the following structured query. The search was limited to studies published between 2016 and 2026 and restricted to English-language publications.

TITLE-ABS-KEY ("sachet water" OR "packaged drinking water" OR "pure water" OR "drinking water quality" OR "water safety")

AND ("plastic waste" OR plastic* OR "plastic pollution" OR "environmental pollution" OR "waste management")

 AND ("health effects" OR "public health" OR "waterborne diseases" OR "environmental health" OR "disease risk")

 AND ("Nigeria" OR "sub-Saharan Africa")

**African Journals Online (AJOL)**

The search was carried out using the search string on the 25^th^ of April 2026 and only studies written in English were considered.

(sachet water OR pure water OR packaged water AND Nigeria AND health OR environment OR pollution OR plastic waste)
